# What Is the Best Predictor of Phenobarbital Pharmacokinetics to Use for Initial Dosing in Neonates?

**DOI:** 10.3390/pharmaceutics13030301

**Published:** 2021-02-25

**Authors:** Martin Šíma, Danica Michaličková, Ondřej Slanař

**Affiliations:** 1 Department of Pharmacology, First Faculty of Medicine, Charles University, 110 00 Prague, Czech Republic; danica.michalickova@lf1.cuni.cz (D.M.); ondrej.slanar@lf1.cuni.cz (O.S.); 2 General University Hospital in Prague, 128 00 Prague, Czech Republic

**Keywords:** phenobarbital, pharmacokinetics, neonates, asphyxia, dosing

## Abstract

Phenobarbital is a first-line treatment of various seizure types in newborns. Dosage individualization maximizing the proportion of patients with drug levels in therapeutic range or sufficient treatment response is still challenging. The aim of this review was to summarize the available evidence on phenobarbital pharmacokinetics in neonates and to identify its possible covariates suitable for individualization of initial drug dosing. Several covariates have been considered: body weight and height, body surface area, gestational and postnatal age, laboratory parameters of renal and hepatic functions, asphyxia, therapeutic hypothermia, extracorporeal membrane oxygenation (ECMO), drug interactions, and genetic polymorphisms. The most frequently studied and well-founded covariate for the estimation of phenobarbital dosing is actual body weight. Loading dose of 15–20 mg/kg followed by a maintenance dose of 3–5 mg/kg/day seems to be accurate. However, the evidence for the other covariates with respect to dosing individualization is not sufficient. Doses at the lower limit of suggested range should be preferred in patients with severe asphyxia, while the upper limit of the range should be targeted in neonates receiving ECMO support.

## 1. Introduction

Neonatal seizures belong among the most common serious neurological disorders worldwide [1]. The incidence of neonatal seizures is estimated between 0.7 to 2.7 per 1000 live term births and increases by two orders of magnitude to 57.5–132 per 1000 live births in preterm neonates [1]. Although there are several anti-seizure drugs available, phenobarbital still remains the first-line agent for the treatment of neonatal seizures [2]. The drug has several favorable features that include undisputed efficacy against a broad spectrum of seizure types, low risk of serious acute adverse drug reactions, multiple pathways involved in the drug elimination as well as availability of parenteral drug formulations and low cost [2]. As suggested by pre-clinical evidence, phenobarbital could have synergistic neuroprotective effects when applied with therapeutic hypothermia [3], which is now considered a standard management for term newborns with moderate to severe encephalopathy [4]. However, long-term outcome benefits have not been fully elucidated on the clinical level yet. Few reports have indicated no improvement of short-term neurodevelopmental outcomes in infants treated for neonatal seizures [5]. On the other hand, phenobarbital also displays several undesirable characteristics that limit its clinical utility. First, there is a significant interpatient variability in the treatment response, which has been confirmed in the clinical studies. Substantial subpopulations of newborns do not respond adequately to phenobarbital treatment and it is not possible to predict inadequate level of responsiveness a priori [6]. Furthermore, there are concerns that phenobarbital may negatively impact psychomotor development and neurological outcomes [7]. This issue has not been specifically addressed for the indication of neonatal seizures. However, a few relatively small studies have indicated the possibility that phenobarbital may affect long-term neurodevelopmental outcomes if the drug was administered either in early childhood for the treatment of febrile seizures or prenatally in gestational medication of their mothers [8,9,10].

## 2. Phenobarbital Pharmacokinetics

Phenobarbital can be administered intravenously, intramuscularly, rectally, or perorally [11]. Summary of the product characteristics states that there is almost complete absorption with T_max_ of 0.5–4 h after oral administration in adults, while only 48.9% bioavailability was reported in neonates [12]. Phenobarbital distribution in the body is characterized by a volume of distribution (Vd) that ranges between 0.48 and 1.56 L/kg in neonates [11,13]. The drug is 40–60% bound to plasma proteins in older children and adults [14], but two- to four-fold less in neonates [15]. The degree of protein-binding subsequently increases as a function of age [16]. Elimination (metabolism and excretion) is characterized by drug clearance (CL). Mean phenobarbital CL values range from 0.0021 to 0.0076 L/h/kg in neonates, which is (together with Vd) reflected in the mean t_1/2_ values of 82–298 h [11,13]. About 25% of drug dose administered is excreted unchanged via urine, while its major proportion is metabolized, principally by oxidation catalyzed by 2C9 enzyme of cytochrome P450 (CYP) with minor contributions of CYP2C19, CYP2E1, and N-glucosidation [17]. Phenobarbital displays pharmacokinetics linearly related to the dose administered [18]. 

Loading and maintenance doses of phenobarbital can be calculated from its Vd and total CL, respectively [19]. In clinical practice, the treatment is usually initiated by intravenous loading dose of 20 mg/kg. If seizures persist, additional bolus doses of 5–10 mg/kg can be administered at 20–30 minutes intervals up to a total dose of 40 mg/kg. Maintenance doses of 3–4 mg/kg/day are commenced 12–24 h after loading dose [20]. However, various studies in the past have used considerably variable dosing schemes using loading doses between 7–20 mg/kg and maintenance doses between 1.3–7.5 mg/kg [21,22,23]. Routine therapeutic drug monitoring (TDM) is recommended during phenobarbital treatment to reach and maintain drug levels in the target therapeutic range, since high pharmacokinetic variability has been reported [24]. Despite the drug being used since 1912, there is no clear consensus on the optimal therapeutic levels to be attained, although phenobarbital levels between 10 and 40 mg/L most likely represent favorable drug exposure. Jalling estimated the therapeutic range of phenobarbital concentration when convulsions ceased of 12–30 mg/L [25], while other studies targeted at a range of 10–30 mg/L [26,27], 20–25 mg/L [28,29], or 15–40 mg/L [22,23,30,31]. TDM-based dose adjustment is feasible only after pharmacotherapy has been introduced, while relatively wide range of doses can be used at the beginning of therapy. Therefore, the identification of suitable covariates for phenobarbital pharmacokinetics allowing dosage individualization with subsequently increased proportion of patients attaining drug levels in the target therapeutic range could be beneficial.

The aim of this review was to summarize the available evidence on phenobarbital pharmacokinetics in neonates and to identify the possible covariates suitable for individualization of initial drug dosing in a neonatal population.

## 3. Methods

A literature search was conducted in PubMed and Web of Science databases up to December 2020 using the following keyword search combinations: neonate or newborn + phenobarbital + pharmacokinetics or dosing. The search was limited to articles published in English and human subjects only. Publications that described phenobarbital pharmacokinetics only with no focus on possible pharmacokinetic and/or dosing covariates were also excluded.

## 4. Results

**Figure 1:**
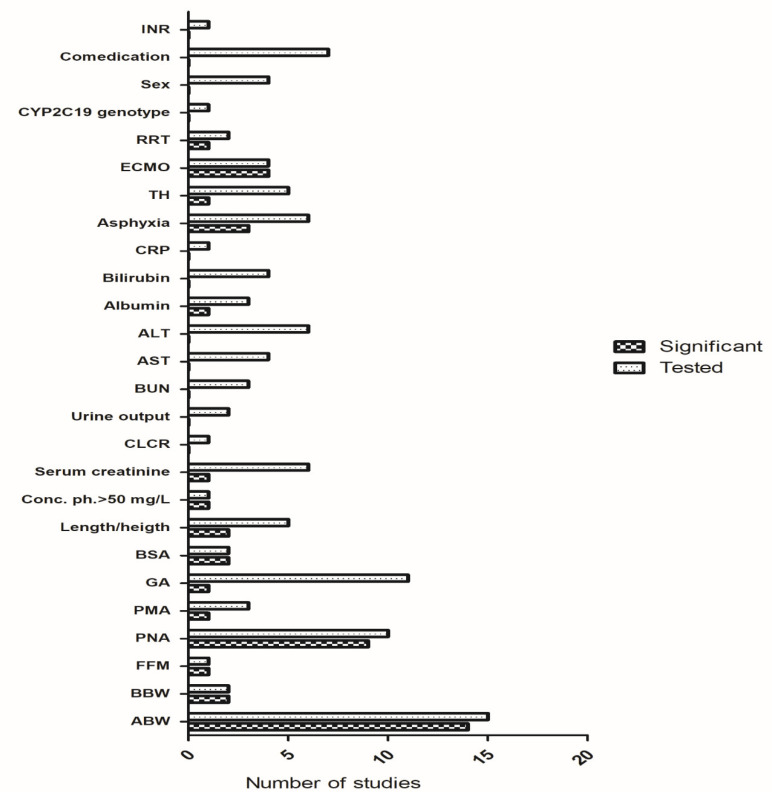
Tested and significant covariates reported in the studies. Abbreviations: ABW = actual body weight, AST = aspartate transaminase, ALT = alanine transaminase, BBW = birth body weight, BSA = body surface area, BUN = blood urea nitrogen, CLCR = creatinine clearance, CRP = C-reactive protein, FFM = fat-free mass, GA = gestational age, ECMO = extracorporeal membrane oxygenation, INR = international normalized ratio, PNA = postnatal age, PMA = postmenstrual age, RRT = renal replacement therapy, TH = therapeutic hypothermia.

In total, 189 studies were found (1967–2020) using the broad search criteria. Twenty-two original articles fit the purpose of this review. Covariates of phenobarbital pharmacokinetics and suggested dosing based on observed relations are summarized in Table 1 and Table 2 respectively. The summary of the tested and significant covariates for phenobarbital pharmacokinetics are outlined in Figure 1.

### 4.1. Covariates of Phenobarbital Pharmacokinetics

#### 4.1.1. Demographics

The most frequently considered covariates for phenobarbital pharmacokinetics in neonates were actual body weight (ABW), gestational, and postnatal age (Table 1 and Table 2).

Some studies have indicated these demographic descriptors as significant covariates for phenobarbital CL [26,32,34]. In addition, Touw et al. also described an association of height and body surface area with Vd and CL, respectively [26]. However, other studies have indicated rather inconsistent data, making conclusions on valid covariates for the drug dosing difficult. We have previously noticed an upward relationship between Vd and ABW, height, and body surface area, whereas CL was not associated with either demographic or clinical features [33]. Pitlick et al. observed no correlation between Vd and gestational age, while CL increased with postnatal age during the first month [35]. Grasela et al. showed that neither Vd nor CL was affected by gestational age [37]. Gilman et al. found no correlation between half-life and either gestational or postnatal age [21]. The study of Völler et al. presented birthweight and postnatal age as the best predictors for maturation of phenobarbital CL and ABW as a predictor for Vd [38]. Moffett et al. showed that significant covariates included fat-free mass (FFM) and postmenstrual age on CL, and FFM and postnatal age on Vd across the pediatric age populations [39]. Back et al. proposed a population nonlinear mixed effect pharmacokinetic, modeling size and maturation functions as covariates of phenobarbital dispositions [51]. In neonates and young infants, both size and maturation functions application was more effective for pharmacokinetic analysis than when only size function was considered. Similar methods and findings have been shown by Thibault et al., where ABW and postnatal age were found as covariates of CL, while ABW predicted phenobarbital Vd [36]. However, this mixed effect approach is relatively exacting and therefore is unlikely to find an application in daily practice.

#### 4.1.2. Laboratory Parameters

No relationships have been observed between laboratory markers of liver functions (total bilirubin, aspartate aminotransferase, alanine aminotransferase, international normalized ratio) and phenobarbital disposition [33,34,39]. In contrast, a recent study using a nonlinear mixed effect approach has described that albumin increases phenobarbital Vd [36]. From the laboratory markers of renal functions, levels of serum urea, serum creatinine, and blood urea nitrogen (BUN) were tested. Urea level was not found to be a significant covariate for the phenobarbital CL. Additionally, creatinine was also not found to be a significant descriptor of the phenobarbital CL variability [33,45,50], while Moffett et al. described a relationship between serum creatinine and phenobarbital CL [39]. As inflammation can influence CYP450 enzyme activity, the C-reactive protein (CRP) level was also tested as a predictor of phenobarbital CL in the critically-ill neonates undergoing ECMO (47), but no relation was found.

#### 4.1.3. Asphyxia

The impact of asphyxia has also been studied, but with contradictory results. Gal et al. reported CL reduction in asphyxiated neonates [40,52], while Grasela et al. noticed no effect on CL, while increased Vd was noted in the presence of asphyxia [37]. Pokorna et al. presented severity of asphyxia as a covariate of phenobarbital CL in patients undergoing therapeutic hypothermia [43], but no effect of asphyxia and its severity on the drug pharmacokinetics was shown in a relatively similar patient population using a population pharmacokinetic modeling approach [48]. 

#### 4.1.4. Therapeutic Modalities

Therapeutic modalities potentially affecting phenobarbital pharmacokinetics that have been studied are therapeutic hypothermia, renal replacement therapy (RRT), and extracorporeal membrane oxygenation (ECMO). Shellhaas et al., van den Broek et al., and Favie et al. have not identified any impact of hypothermia on the phenobarbital disposition [34,41,42]. Although Filippi et al. stated that phenobarbital administered to newborns under whole body hypothermia resulted in higher plasma concentrations and longer half-lives than expected in normothermic newborns, this study did not contain any normothermic control group, making comparison of the pharmacokinetics between the hypo- and normo-thermic neonates impossible [30]. Thus, the effect of therapeutic hypothermia does not seem to be clinically relevant for phenobarbital dosing. A recent study has found that interaction of severity of asphyxia and hypothermia is associated with a clinically relevant reduction of phenobarbital CL, suggesting the potential relevance of disease characteristics beyond hypothermia itself [43].

Pokorna et al. observed increased phenobarbital CL in neonates receiving ECMO support, while Vd was not significantly different compared to neonates without ECMO [44]. These observations are consistent with the necessity of higher doses in ECMO patients described by Dillman et al. [53]. Thibault et al. reported the effect of ECMO therapy on phenobarbital Vd in neonates after congenital heart surgery, resulting in the need for a higher loading dose, but the drug CL was not affected [36]. In another study, the same research group found a 6-fold increase of phenobarbital CL in neonates and infants undergoing continuous veno-venous hemodiafiltration (CVVHDF) compared to the neonates and infants without CVVHDF. Additionally, the authors found no impact of ECMO on phenobarbital Vd. When analyzed phenobarbital levels before, during, and after ECMO, Michaličková et al. observed that phenobarbital CL linearly increased with time during the ECMO phase, while in the post-ECMO phase, CL initially decreased and subsequently increased slowly, which was likely driven by maturation [45]. Moreover, the authors found no impact of ECMO on phenobarbital Vd. Thus, data indicated that higher phenobarbital doses are needed during ECMO, however, the particular dosing recommendation varied. 

#### 4.1.5. Drug Interactions and Genetic Polymorphisms

Impact of the co-medication of several drugs on phenobarbital pharmacokinetics has been investigated repeatedly [37,41,42,46,53]. No significant effect of co-administered dopamine, dobutamine, norepinephrine, phenytoin, sufentanil, midazolam, tramadol, or furosemide was observed in short-term concomitant treatment [46]. Michaličková et al. also did not observe any effect of diuretics and inotrope use on phenobarbital CL in critically-ill neonates undergoing ECMO [41]. Although Moffett et al. concluded that midazolam, phenytoin, and pantoprazole significantly affected phenobarbital CL [39], this finding has been questioned as a chance finding only [54,55]. The cytochrome P450 2C19 genotype did not also affect phenobarbital pharmacokinetics in neonates and infants [47].

### 4.2. Covariate-Based Phenobarbital Dosing

The routinely used phenobarbital dosing in neonates is based on body weight and consists of loading dose of 15–20 mg/kg followed by maintenance dose of 3–5 mg/kg per day. Several body weight-based dosing regimens or nomograms have been described in studies included in this review. The intersection of these findings well corresponds to the above-mentioned dosing routines.

## 5. Discussion

Phenobarbital has been introduced in clinical practice for more than 100 years as the first efficacious organic anti-seizure drug. It still belongs among the most potent medications with relatively modest acute toxicity, however, its sedative effects, tendency to disturb behavior in children, and development of tolerance and dependence limit the clinical utility of the drug in all age groups of pediatric patients. The complex clinical utility is a result of very broad neurochemical and neurophysiological changes induced by the drug. However, their clinical relevance are currently not fully elucidated. As a consequence, the relationship between the pharmacokinetics of the drug and its efficacy/safety is even more difficult.

Ouvrier et al. described that an earlier achievement of target therapeutic serum concentration in newborns, who were given intravenous and high intramuscular loading doses, resulted in earlier control of serial seizures compared with the group with no loading dose [56]. Several earlier studies recommended different dosing ranges (corresponding to different serum target concentrations). Ouvrier recommended loading and maintenance doses of 15 mg/kg and 6 mg/kg, respectively. The respective dosing proposed by other studies has been reported in the ranges of 8–20.5 mg/kg and 1.3–7.5 mg/kg. This recommendation of a relatively wide dosing range cannot be only explained by the different target concentration ranges used in the different studies, in which most of the authors aimed at the pharmacokinetic targets fitting between 10 to 40 mg/L. The target ranges of 15–40 mg/L and 10–30 mg/L were the most commonly applied.

The drug was introduced well before the current strict guidelines and requirements for drug development went into force, therefore the knowledge on pharmacokinetics and optimal dosing has been established during routine use. Moreover, neonates display generally increased intra- as well as inter-individual variability of pharmacokinetic properties for many drugs due to the rapid developmental changes. Phenobarbital pharmacokinetics is further likely to be affected by other pathophysiological processes in the disease state that can be connected to general health status (e.g., asphyxia), altered drug elimination capacities (renal impairment, decreased liver functions), decreased cardiac output, or other changes in hemodynamics [57]. Advanced life support management (e.g., ECMO) or renal replacement therapy may represent another factor contributing to the complex issue. Therefore, even after a long history of clinical use of phenobarbital, the selection of optimal dosing for an individual neonate is challenging and many possible covariates have been tested.

Among the demographic parameters, ABW was the most frequently reported significant covariate for phenobarbital pharmacokinetic parameters, although even this routinely used parameter for dosing normalization was found as a significant covariate for phenobarbital Vd and CL in 14 and 10 studies, respectively. For the other demographic parameters or body size descriptors, the evidence is weaker and has been described in isolated studies only.

ABW has been shown to be more predictive for phenobarbital CL than height, body surface area, or gestational age [26]. This observation seems plausible considering that ABW mirrors both prenatal and postnatal maturation and also reflects the general prosperity of neonates. The drug CL increased with increasing ABW, which can be attributed to developmental changes of the rapidly growing subjects occurring in parallel with the maturation of elimination functions. Thus, it can be assumed that ABW serves as a marker for maturation in this patient population. In contrast, gestational and postnatal age represent solely prenatal and postnatal maturation, respectively. Other body size descriptors (ideal body weight, adjusted body weight, FFM, lean body mass, and body surface area) are used, especially in adult obese populations, for dosing estimation of hydrophilic drugs [58,59], but their use in neonates, in whom ABW is not yet distorted by obesity, is not justified. Moffett et al. have reported FFM to be a significant covariate for both Vd and CL, however, this parameter is not easily applied in clinical settings. Actually, the formula for FFM calculation used in the aforementioned paper may not be appropriate for this age group, since it has not been developed or validated for children younger than three years of age [60]. Therefore, we do not recommend using FFM as a parameter for phenobarbital dosing prediction in neonates.

Moreover, ABW has been identified as the best predictor of dosing for some other drugs in a pediatric population [61,62,63]. Therefore, it is widely used in clinical settings.

Although allometric scaling based on body weight to extrapolate PK parameters from adults to a pediatric population is frequently used in studies [12,34,41,55], there is no evidence for one unique allometric exponent in this population as its use leads to increasingly worse predictions with decreasing age [64].

There was inconsistent impact of hepatic and renal function status on phenobarbital pharmacokinetics, which can be explained by the involvement of multiple elimination pathways in phenobarbital metabolism and excretion. In the case when the capacity of one of the elimination pathways decreases, the deficit can be compensated by the other one. Only one study found a significant correlation between renal function (represented as serum creatinine) and phenobarbital CL [55]. Additionally, a study in neonates after heart surgery reported that phenobarbital Vd decreased with increasing albumin values. However, the physiological plausibility of such a finding is not fully clear. Considering that 28–36% of the total phenobarbital concentrations are bound to the proteins in neonates, it is not likely that even severe albuminemia causes significant changes in Vd [65]. Finally, serum albumin and creatinine values do not represent reliable markers of liver and kidney functions in children [66,67].

The impact of genetic polymorphisms on phenobarbital pharmacokinetics in neonates has been studied only in the study by Lee et al. [47]. While there are other pharmacokinetic studies in adults studying the possible effect of CYP2C9 or CYP2C19, the results have been discrepant or difficult to interpret due to the exclusion of the possible effect of CYP2C9 polymorphism in the analysis for CYP2C19. Moreover, the extrapolation between adult and neonatal populations may be limited due to extensive and non-linear postnatal phenotypic ontogeny. However, due to the multiple pathways of the drug elimination involved, the possible effect of these polymorphisms is expected to be minor if any.

Concomitant medication was also tested as a covariate of phenobarbital CL. No significant effect of co-medication (dopamine, dobutamine, norepinephrine, phenytoin, sufentanil, midazolam, tramadol, and furosemide) was observed in short-term concomitant treatment [46]. Michaličková et al. also did not observe the effect of diuretics and inotrope use on phenobarbital CL in critically-ill neonates undergoing ECMO [45]. Moffett et al. reported a decrease in phenobarbital CL with concomitant use of midazolam and phenytoin, and an increase if pantoprazole was used [39]. However, this finding has been questioned [54,55]. Midazolam is a substrate of the CYP3A4 isoenzyme, which is not associated with phenobarbital metabolism [68]. Pantoprazole, unlike other proton pump inhibitors, has no or little potential for enzyme induction [69]. Therefore, co-medication does not seem to be a significant descriptor of phenobarbital pharmacokinetics.

Current state-of-the-art modalities such as ECMO, therapeutic hypothermia, and RRT introduce additional factors, possibly altering the pharmacokinetics of the drug. The majority of the available data indicate that hypothermia most likely does not represent a clinically important covariate for drug dosing [34,41,42,49]. On the other hand, ECMO treatment likely affects the pharmacokinetics of phenobarbital, although the available evidence is variable and thus insufficient to determine the magnitude of its effect. Michalickova et al. found a time-dependent linear increase in CL, but not in Vd, which might be explained by the scarce sampling at early time-points of phenobarbital administration [45]. Conversely, Thibault et al. reported the increasing effect of ECMO therapy on phenobarbital Vd, but no effect on CL [36]. In another larger group of patients, the same research group found no effect of ECMO on Vd, but reported a 6-fold increase of phenobarbital CL in patients undergoing CVVHDF compared to the patients without CVVHDF [50]. Given the small number of patients included in these analyses, larger studies are needed to elucidate the pharmacokinetic changes induced by ECMO to recommend phenobarbital dosing in this specific clinical setting. It seems that the upper limit of the pharmacokinetic target range should be considered for neonates receiving ECMO support until further evidence allows for the elaboration of a more precise dosing strategy in this patient subpopulation.

The evidence on the effect of hypoxia on phenobarbital pharmacokinetics/dosing is largely inconsistent. Perinatal asphyxia is associated with impaired hepatic function, which could result in reduced metabolism and CL of hepatically metabolized drugs such as phenobarbital [70]. Indeed, early studies directly comparing asphyxiated and non-asphyxiated patients showed decreased CL in newborns with asphyxia [40,52]. Recent studies, however, did not confirm these observations [34,48]. Additionally, only one study found a 13% increase in Vd of phenobarbital in neonates with 5-min Apgar score less than 5 [37], while no relationship between Vd and Apgar score has been seen by other authors [34,48]. As phenobarbital is a weak acid with a pKa of 7.3, alterations in blood pH can affect the Vd of phenobarbital, with the decrease in blood pH leading to a significant increase in Vd of phenobarbital. Metabolic acidosis likely explains the association of Vd with low 5-min Apgar score.

High heterogeneity in the findings addressing the importance of asphyxia on phenobarbital pharmacokinetics is likely explained by the differences in the data analysis methods (e.g., pharmacokinetic modeling approach use or not) [43,48] and the use of variable and controversial definitions of asphyxia. Namely, the reported criteria of asphyxia are Apgar scores, umbilical pH level, and base excess. Apgar scores suffer from poor sensitivity and specificity partly due to their subjective nature, leading to high levels of inter-observer variability [71]. Therefore, this overall inconsistency makes impossible any firm conclusions for phenobarbital dosing with respect to asphyxia, however, as a pre-cautionary measure, it seems rational to prefer doses at the lower limit of the suggested range for neonates with severe asphyxia to prevent possible overdosing.

We acknowledge that this paper is a critical review of the literature and does not represent a systematic review. Therefore, the inherent limitations of our review should be considered. Moreover, our work summarizes evidence, which is generally insufficiently robust due to the small study sample sizes in the primary reports, highly heterogenous methodologies used, and inconsistent enrolment criteria. However, neonates treated with phenobarbital are a very specific and vulnerable patient population that receive advanced intensive care. Therefore, any data that could improve drug dosing and management are of importance, even if they may not be optimal in the view of current criteria for evidence based medicine.

## 6. Conclusions

There are very heterogeneous observations with respect to possible covariates that could alter phenobarbital pharmacokinetics and which could be subsequently used for dosing individualization. The causes of this heterogeneity lie in the relatively limited study sample sizes, heterogeneous patient study populations, variable methodologies used, and relatively long-time span over which the studies have been conducted, which affects diagnostic and therapeutic procedures.

A clear conclusion that can be drawn from the available literature is that there is no single descriptor that is indisputably the best estimate for phenobarbital dosing in neonates. However, the most frequently described and well-founded covariate for estimation of phenobarbital dosing is ABW. A loading dose of 15–20 mg/kg, followed by a maintenance dose of 3–5 mg/kg/day seems to be appropriate. Furthermore, doses at the lower limit of the suggested range should be preferred in patients with severe asphyxia, while the upper limit of the range should be targeted in neonates receiving ECMO support and RRT.

TDM after the initiation of the treatment is still necessary to guide the dosing in individual patients due to the large and unexplained part of pharmacokinetic variability in neonates. Finally, it is important to emphasize that these dosing recommendations are made only according to the pharmacokinetic considerations and have not been confirmed in properly designed prospective clinical trials. Therefore, future pharmacokinetic analyses should be properly powered and prospectively designed. Optimally, they should also elucidate the relationship between pharmacokinetic variability and the efficacy/safety of phenobarbital.

## Figures and Tables

**Table 1:** Patient characteristics and the main findings of the studies evaluating the covariates of phenobarbital pharmacokinetics.

Population	Location	Considered Covariates	Observed Relationships—Vd	Observed Relationships—CL	Reference
Term and preterm neonates (*n* = 19; 10 preterm, 9 term)	The Netherlands	ABW, length, BSA, GA	Vd increases with increasing ABW, length, BSA, and GA	CL increases with increasing ABW, length, BSA, and GA	Touw et al. [26]
Neonates and infants (*n* = 70)	Japan	ABW, GA, PNA, postconceptional age, gender	Vd increases linearly with increasing ABW	CL increases linearly with increasing ABW and PNA; CL decreases nonlinearly with increasing phenobarbital serum concentration >50 mg⁄L	Yukawa et al. [32]
Term neonates with moderate to severe asphyxia (*n* = 36)	Czech Republic	ABW, length, BSA, GA, serum creatinine, creatinine CL estimation according Schwartz formula, total bilirubin, ALT, AST, INR, Apgar scores, umbilical cord arterial blood pH, base excess	Vd increases with increasing ABW, length and BSA	CL is not affected	Sima et al. [33]
Asphyxiated neonates (*n* = 39; 20 hypothermia, 19 normothermia)	USA	Therapeutic hypothermia use, ABW, GA, PNA, Apgar score, AST, ALT	Vd increases linearly with increasing ABW	CL increases with increasing ABW and PNA	Shellhaas et al. [34]
Neonates (*n* = 16)	USA	GA, PNA	None	None	Gilman et al. [21]
Preterm and term neonates (*n* = 8)	USA	GA, PNA	None	Half-life decrease (implying increase of CL) with increasing PNA	Pitlick et al. [35]
Neonates after congenital heart surgery (*n* = 37)	USA	ABW, PNA, PMA, ECMO, and RRT use, albumin, ALT, BUN, serum creatinine, co-medication (pantoprazole, midazolam, [fos]phenytoin), surgery-related data (primary cardiac anomaly, procedure performed, CPB times)	Vd increases with ECMO for 21%; increases linearly with increasing ABW and decreased with increasing albumin values	CL increases with increasing PNA and ABW	Thibault et al. [36]
Preterm and term neonates (*n* = 59)	USA	Asphyxia, GA, gender, duration of therapy	Asphyxia increases Vd by 13%	CL is not affected	Grasela et al. [37]
Preterm and term neonates (*n* = 53);	The Netherlands	Birth weight, ABW, height, PNA, PMA, GA, sex, liver and kidney function, Apgar score	Vd increases linearly with increasing ABW	CL increases linearly with increasing birthweight and PNA	Voller et al. [38]
Pediatric patients (<19 years) (*n* = 355; 42.5% neonates, 7.6% were >30 days of age and <2 years of age)	USA	ABW, height, GA, PNA, PMA, core body temperature, serum creatinine, BUN, AST, ALT, urine output over the prior 12 hours, and co-medication	Vd linearly increases with increasing FFM; Vd decreased with increasing PNA	CL increases with increasing FFM and PMA; CL decreased with increasing creatinine, phenytoin and midazolam decreases CL (by 40% and 24%, respectively), pantoprazole increases CL by 25%	Moffett et al. [39]
Asphyxiated and nonasphyxiated neonates (*n* = 18; 11 asphyxiated, 7 non-asphyxiated)	USA	Asphyxia	Vd is not affected	Asphyxia reduces phenobarbital CL	Gal et al. [40]
Asphyxiated term neonates (*n* = 31)	The Netherlands	Therapeutic hypothermia (body temperature), ABW	Vd increases linearly with increasing ABW	CL increases with increasing ABW	van den Broek et al. [41]
Neonates (*n* = 113)	The Netherlands	Therapeutic hypothermia	Vd increases linearly with increasing ABW	CL increases with increasing ABW	Favie et al. [42]
Asphyxiated term neonates (*n* = 40; 26 hypothermia, 14 normothermia)	Czech Republic	ABW, GA, co-medication with phenytoin, therapeutic hypothermia, asphyxia and its severity	Vd correlates with ABW	CL correlates with ABW and GA	Pokorná et al. [43]
Neonates and infants received ECMO (*n* = 16; 7 neonates, 9 infants)	Czech Republic	ECMO	None	CL increases during ECMO	Pokorná et al. [44]
Neonates received ECMO (*n* = 13)	Czech Republic	ABW, PNA, ECMO and ECMO set up characteristics, co-medication (inotropes, diuretics), serum creatinine, serum urea, serum albumin, total and direct bilirubin, CRP, blood pH, AST, ALT, and urine output; concomitant continuous renal replacement therapy	Vd increases linearly with increasing ABW	CL increases linearly with increasing PNA and birthweight; ECMO increases CL in a linear time-dependent way	Michaličková et al. [45]
Asphyxiated term neonates (*n* = 37)	Czech Republic	Co-medication: dopamine, dobutamine, norepinephrine, phenytoin, sufentanil midazolam, tramadol, and furosemide	None	None	Sima et al. [46]
Neonates and infants (age range of 8 days to 6 months) (*n* = 44)	Korea	CYP2C19 genotype: 991A>G (I331V), 681G>A (P227P), and 636G>A (W212X)	Vd increases with increasing ABW	CL increases with increasing ABW and PNA	Lee et al. [47]
Neonates, GA 39-40 weeks (*n* = 50,)	Czech Republic	Age, ABW, sex, concomitant medications, Apgar scores, serum creatinine, lactate, base excess,	Vd increases linearly with increasing ABW	None	Pokorna et al. [48]
Asphyxiated neonates (*n* = 19) treated with TH	Italy	Apgar scores, serum creatinine, lactate, base excess, BBW, pH	None	None	Filippi et al. [30]
Asphyxiated neonates (*n* = 113) treated with TH	The Netherlands	GA, TH (body temperature), PNA, ABW	Vd increases linearly with increasing ABW	CL increases with ABW	Favie et al. [49]
Neonates and infants undergoing ECMO and RRT (*n* = 35)	USA	ECMO—set up characteristics, concomitant RRT (CVVHDF, CVVH, SCUF), levels of BUN, ALT, creatinine, and albumin; co-medication (pantoprazole, midazolam, and [fos]phenytoin);	Vd increases linearly with increasing ABW	CL increases linearly with increasing PNA and ABW; increases 6 times in CVVHDF	Tribault et al. [50]

Abbreviations: ABW = actual body weight, ALT = alanine aminotransferase, AST = aspartate aminotransferase, BSA = body surface area, BUN = blood urea nitrogen, CL = clearance, CRP = C-reactive protein, CVVH = continuous veno-venous hemofiltration, CVVHDF = continuous veno-venous hemodiafiltration, ECMO = extracorporeal membrane oxygenation, FFM = fat-free mass, GA = gestational age, INR = international normalized ratio, PMA = postmenstrual age, PNA = postnatal age, RRT = renal replacement therapy, SCUF = slow continuous ultrafiltration, TH = therapeutic hypothermia, Vd = volume of distribution, VA = veno-arterial, VV = veno-venous.

**Table 2:** Suggested phenobarbital dosing based on covariates of the pharmacokinetic parameters.

Population	Location	Dosing Covariate	Suggested Dosing	Reference
Term and preterm neonates (*n* = 19; 10 preterm, 9 term)	The Netherlands	ABW	Loading dose: 15.3 mg/kg + 12 mg Maintenance dose: 2.66 mg/kg + 0.72 mg per day	Touw et al. [26]
		Height	Loading dose: 2.64 mg/cm—72 mg Maintenance dose: 0.4 mg/cm—10.8 mg per day	
		BSA	Loading dose: 330 mg/m^2^—7.5 mg Maintenance dose: 54.7 mg/m^2^ + 2.2 mg per day	
		GA	Loading dose: 3 mg/week—57 mg Maintenance dose: 0.55 mg/week—11.5 mg per day	
Term neonates with moderate to severe asphyxia (*n* = 36)	Czech Republic	ABW	Loading dose: 15 mg/kg Maintenance dose: 3 mg/kg/day	Pokorna et al. [33]
Asphyxiated and non-asphyxiated neonates (*n* = 18; 11 asphyxiated, 7 non-asphyxiated)	USA	ABW	Maintenance dose asphyxiated: 1.8 mg/kg/day Maintenance dose non-asphyxiated: 4 mg/kg/day	Gal et al. [40]
Neonates and infants treated with ECMO (*n* = 16; 7 neonates, 9 infants)	Czech Republic	ABW	Loading dose: 15 mg/kg Maintenance dose: 4 mg/kg/day	Pokorna et al. [44]
Neonates (*n* = 37; 12 treated with ECMO)	USA	ABW, ECMO, albumin	Loading dose: 30 mg/kg in neonates on ECMO; 30 mg/kg in neonates with serum albumin ≤3 mg/dL; 20 mg/kg in neonates with serum albumin ≤3.5 mg/dL Maintenance dose: 4–5 mg/kg/day	Thibault et al. [36]
Neonates treated with ECMO (*n* = 13)	Czech Republic	PNA, ABW, ECMO	Loading dose: 20 mg/kg Maintenance dose: 4 mg/kg/day with an increase of 0.25 mg/kg every 12 h during ECMO treatment	Michaličková et al. [45]
Neonates and infants undergoing ECMO and CRRT (*n* = 35)	USA	ABW	Loading dose: 30 mg/kg Maintenance dose: 6–7 mg/kg divided in 2 doses given every 12 h	Thibault et al. [50]
		ABW, CVVHDF	Loading dose: 30 mg/kg Maintenance dose: 40 mg/kg/day administered in divided doses every 6 hours	

Abbreviations: ABW= actual body weight, BSA = body surface area, ECMO = extracorporeal membrane oxygenation, GA = gestational age.

## Data Availability

Data sharing not applicable.
